# Addressing the challenges of reconstructing systematic reviews datasets: a case study and a noisy label filter procedure

**DOI:** 10.1186/s13643-024-02472-w

**Published:** 2024-02-17

**Authors:** Rutger Neeleman, Cathalijn H. C. Leenaars, Matthijs Oud, Felix Weijdema, Rens van de Schoot

**Affiliations:** 1 Department of Methodology and Statistics, Faculty of Social and Behavioral Sciences, Utrecht University, Utrecht, The Netherlands; 2 Institute for Laboratory Animal Science, Hannover Medical School, Hannover, Germany; 3 Department Care and Participation, Trimbos-Institute, Da Costakade 45, 3521 VS Utrecht, the Netherlands; 4 Utrecht University Library, Utrecht University, Utrecht, the Netherlands

**Keywords:** Systematic reviews, Replication, Active learning, Noisily labeled records, ASReview

## Abstract

Systematic reviews and meta-analyses typically require significant time and effort. Machine learning models have the potential to enhance screening efficiency in these processes. To effectively evaluate such models, fully labeled datasets—detailing all records screened by humans and their labeling decisions—are imperative. This paper presents the creation of a comprehensive dataset for a systematic review of treatments for Borderline Personality Disorder, as reported by Oud et al. (2018) for running a simulation study. The authors adhered to the PRISMA guidelines and published both the search query and the list of included records, but the complete dataset with all labels was not disclosed. We replicated their search and, facing the absence of initial screening data, introduced a Noisy Label Filter (NLF) procedure using active learning to validate noisy labels. Following the NLF application, no further relevant records were found. A simulation study employing the reconstructed dataset demonstrated that active learning could reduce screening time by 82.30% compared to random reading. The paper discusses potential causes for discrepancies, provides recommendations, and introduces a decision tree to assist in reconstructing datasets for the purpose of running simulation studies.

**Supplementary Information:**

The online version contains supplementary material available at 10.1186/s13643-024-02472-w.

## Introduction

Systematic reviews and meta-analyses are used to synthesize the available data on specific research topics and are generally viewed as studies with the highest level of evidence [1, 31]. However, the task of screening records for relevance can be extremely time-consuming, as thousands of records often need to be screened [28]. Machine learning-aided tools have been developed to overcome this challenge, including active learning models [32] for screening prioritization [10]. Simulation studies can mimic the screening process to investigate the performance of different models instead of random reading. With such simulation studies, it can be investigated how much work can be saved employing machine learning models compared to random reading and what model works best for what type of data. For a systematic overview of such simulation studies, see [33].

For a simulation study, a fully labeled dataset containing the records screened by the researchers, including all their labeling decisions, is needed. Only with this information can a simulation study mimic the screening process to see if machine-learning models can predict the labels provided by a human. However, according to the PRISMA guidelines for reporting systematic reviews [25], researchers are only expected to report their inclusion criteria, describe all information sources, and report their full search strategy for at least one database. In the updated version of 2020, these criteria were extended to the “full search strategies for all databases, registers, and websites, including any filters and limits used” ([30], p. 1). Unfortunately, from a machine learning perspective, the guidelines do not require researchers to store the fully labeled dataset, and often, only the list of included records is published.

Theoretically, one could say that by replicating the steps reported in the initial paper, it should be possible to reconstruct the initial dataset and apply the labels to the irrelevant records. That is, by exactly reproducing the search queries, using date filters to obtain the initial list of results per query, merging and deduplicating these results, and labeling based on the initial inclusion criteria, one should obtain the same list of records used by the original paper’s authors. In an ideal scenario, a comprehensive tracking dataset would be maintained, documenting the labeling decisions made by each screener at every stage of the process (title and abstract screening, full-text screening, and final inclusion in the meta-analysis). This level of detail would facilitate a more accurate reconstruction of the study’s dataset for future research and verification purposes.

However, in practice, there are several reasons why the initial and reconstructed datasets do not entirely overlap, all outside the influence of the initial authors. A first reason might be institutional access. While searching the same literature databases, it may be that these are accessed via different platforms; for example, if the original authors searched PsycInfo via EBSCOhost and Embase via Ovid, the replication team accessed PsycInfo via Ovid and Embase via Embase.com. Unknown to many, these platforms have some differences, mainly in how the explode function works—only one level down via EBSCOhost, while most other platforms include the entire hierarchy below, explained in, for example, [9]. A second reason might be that it is not always clear which fields are searched with specific syntax via different platforms and whether this changes over time. For example, the [tiab] code for Pubmed searches for text terms in title, abstract, and author-provided key-words stored in the [OT] field, while even many specialists are unaware of the latter. A third reason might be that the internal dictionaries can change over time; new terms are added, terms are removed, the way terms are used can change, and the hierarchy can also be adapted (see Table 2 in [37], for examples). The same applies to classification codes and journal subsets. The fourth reason might be that the databases will correct the occasional errors; if an error was present at the initial search date, corrections will result in differences in the retrieved dataset at a later date [15]. A fifth reason might be that occasionally, papers are retracted (e.g., [3]) , or that literature databases started indexing additional journals or added papers for the new journals retrospectively.

The result of replicating a search is a reconstructed dataset, but due to many small changes in databases, it is unknown whether precisely the identical records are part of this replicated dataset compared to the initial dataset. It may be that additional records are found in the reconstructed dataset that were not part of the initial dataset, and it cannot be ruled out that these records are relevant according to the initial inclusion criteria. Such records are what we call noisy labels, which negatively impact the performance of machine learning models as given labels are assumed to be correct [17]. In theory, one could correct the labels when aiming to replicate a search at a later date by manually checking the database changes for each thesaurus term included in the initial search. In practice, going through all the thesaurus terms within a search is not always viable, as there are many terms within a search, and for each of the terms for each of the databases, the entire hierarchy needs to be checked. An example from the case study: for the Emtree term “psychotherapy”, there are over 50 underlying terms, seven of which contain further underlying terms, and several of those have even more below [13]. Therefore, such a manual process is inefficient, if possible at all.

**Fig. 1:**
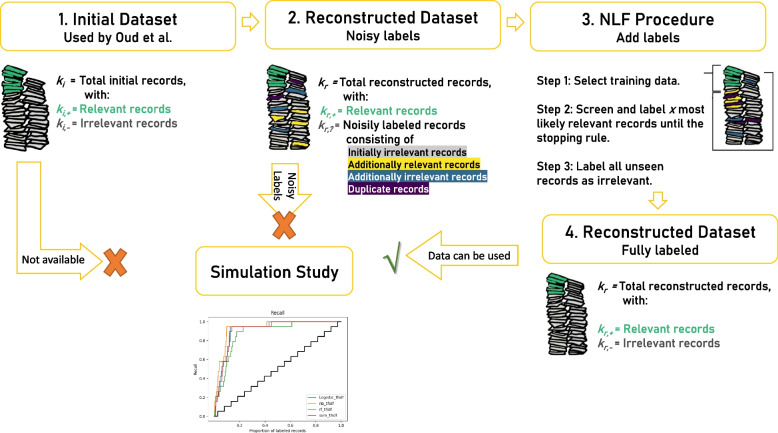
Schematic representation of record types and labeling notations

Thus, the issue is that the initial dataset is unavailable, and the replicated dataset contains noisy labels, but it is unknown what records are noisy. As such, both datasets cannot be used for running a simulation study; see for a schematic overview and definitions in Fig. 1. Therefore, we introduce a Noisy-Label-Filter procedure, which corrects the noisy labels so that the reconstructed dataset can be used for the simulation study. This procedure is designed for the situation if one only has access to the initial search queries and a list of relevant records without access to a fully labeled dataset.

Using a case study approach, in the first part of the paper, we provide a detailed description of our replication, including the methods and techniques used to create the replicated dataset. For the case study, we used a paper about a systematic review of the psychological treatment of Borderline Personality Disorder [29]. It is important to note that Oud et al. adhered to the version of the PRISMA guidelines applicable at the time of their systematic review, which required the provision of a complete search strategy. The reasons for the mismatch are related to several reasons outside the influence of the original authors.

In the second part of the paper, we introduce a solution addressing the mismatch between the reconstructed and the initial dataset with a noisy label filter (NLF) procedure. This procedure can potentially detect additional relevant records with minimal screening effort. We applied the NLF procedure to the case study, and in the third part of the paper, we provide the results of a simulation study comparing the performance of different active learning models with manual screening to find out how much work Oud et al. could have saved if they had used active learning. Finally, in the discussion, we provide a decision tree for dealing with the more generic problem of conducting a simulation study without a fully labeled dataset.

## Reconstructing a dataset

###  Methods

In this section, we describe the process of replicating the process of Oud et al. [29]. The aim was to reconstruct the initial dataset as accurately as possible. The following components were available:

1. A complete list of search queries for all databases and a search history file, as stored in the supplementary material.
2. The number of records identified for each search query.
3. The total number of screened records for the initial dataset as reported in the PRISMA flowchart, denoted by *k*_*i*_ with ‘ + ’ indicating the number of included records and ‘*-‘* for excluded records. In Oud et al. *k*_*i*_ = 1013, of which *k*_*i,*+_  = 21 were labeled as relevant, and *k*_*i,-*_ = 992 as irrelevant.
4. A list of the references that were included in the meta-analyses (see Table 1 in Oud et al.).

**Table 1:** Overview of the number of queries whose results were exported to the initial and reconstructed database

	Results in the initial query	Results in the reconstructed query
PsycINFO	533	566
MEDLINE	444	486
The Cochrane Library	14	0
CinaHL	116	201
Embase	303	290
Total	1410	1543

We first used component 1 to reproduce the initial search queries. With one exception, we searched the same search engines as the initial study (Embase, The Cochrane Library, PsycINFO, MEDLINE, CinaHL). We did not search The Cochrane Library database because of viability, search date filtering problems, and expected record overlap. This did not result in missing relevant records, as described in the discussion. Also, Due to differences in institutional access, the same databases had to be searched via different vendors/apps. Additional file 1: Appendix 1 provides a detailed description of the process we used for replicating each search query, and the field codes and search syntax per platform can be found in Table A.1.

The information from components 2 and 3 was used to check whether the number of records we found with the other databases matched the initial numbers as listed in the search history file, see Tables A.2-A.5 in Additional file 1:  Appendix 1. We encountered several issues when trying to replicate the exact queries used in the initial study. Some initial queries yielded an error message, while others retrieved fewer or more records than the initial queries. Simple syntax changes could solve some of the errors and differences. For example, the EBSCO near/3 function was converted to ‘adj4’ in Ovid.

For each problematic search query, we describe our pragmatic alternative. Detailed information about the initial search terms, the reconstructed search terms, the number of results per query, and differences between the initial and replication can be found in Additional file 1:  Appendix 1. The reconstructed dataset (denoted by *k*_*r*_) can be found on the Open Science Framework (OSF); [27].

### Results

Table 1 summarizes the number of papers that were exported to our Zotero database, available on the OSF [27]. As shown in Table 1, the reconstructed queries produced more results for each database than the initial one.

#### Creating the reconstructed dataset

After exporting all the data from the search engines into the open-source reference manager Zotero, the reconstructed data contained *k*_*r*_ = 1543 records. We then verified that the initial relevant records were present in this database, which was the case, hence *k*_*i,*+_  = *k*_*r,*+_. Then, we deduplicated the dataset as described in detail in Additional file 1:  Appendix 2. In the first deduplication phase, 480 duplicates were automatically detected and deleted based on title, abstract, and DOI, leaving a database of 1062 records. In the second phase, we manually reviewed the relevant studies to look for duplicates that were not caught in the first phase. Some records appeared to be not exact duplicates (with completely identical titles, abstracts, and DOIs) but could be considered equivalent because they report on the same data or study. These can often result from different indexing practices across databases, slight variations in titles or abstracts, or even multiple reports of the same study results. We aimed to align with the process and exclusion decisions made by the original authors, Oud et al. ensuring that our reconstructed dataset closely mirrors the one used in their systematic review. Therefore, we have adopted the same rigorous approach to identify and delete these, so-called, semi-duplicates through a manual review process, as detailed in Additional file 1:  Appendix 2 (*k* = 9). This step was essential to maintain consistency with the original study and to ensure the accuracy of our simulation results.

After the two stages of deduplication, our version of the reconstructed database included *k*_*r*_ = 1053 records with 21 relevant records, except for the initially included article by [3], which was retracted after Oud et al.’s study was performed, so *k*_*r,*+_  = 20. The remaining *k*_*r,?*_ = 1033 records are so-called, records with noisy labels.

## Noisy label filter procedure

The reconstructed dataset poses a challenge for running simulation studies due to unknown labels for the *k*_*r,?*_ = 1033 labels. This section explains why naively labeling these records as irrelevant is not a straightforward solution. We introduce the Noisy Label Filter (NLF) procedure to correct the noisy labels, and we apply the NLF procedure to the case study.

### Background

We do not have a list of initially excluded records to compare with the reconstructed dataset, so whether the initial and reconstructed datasets contain precisely identical records is unknown. We only know whether the relevant records are part of the reconstructed dataset because these are listed in Table 1 in the meta-analyses. There are four possible types of records in the reconstructed dataset for *k*_*r,?*_ see also Fig. 1:

1. Initially irrelevant records: these records are classified as irrelevant by the original paper’s authors.
2. Additional relevant records: these are records that were not part of the initial dataset but should have been labeled as relevant if they had been screened in the initial study. This is the most problematic type if all 1033 records would be labeled as ‘irrelevant’. The presence of falsely labeled irrelevant records could confuse the model, causing incorrect categorization and unreliable results.
3. Additional irrelevant records: these are new records found in the reconstructed search that should have been labeled as irrelevant if found in the initial study.
4. Duplicates: these are records that were initially removed from the data during deduplication but were left undeleted in the reconstructed database due to differences in the deduplication method.

We cannot differentiate between these four categories without access to the entire list of initial records. As a result, any records in the reconstructed dataset but not included in the initial dataset should be considered noisy. Labeling all the noisy labels as irrelevant may seem a simple solution. However, the problem is that we might miss possible additional relevant records.

### Step-by-step procedure

To address the potential problem of additional relevant records being labeled as irrelevant in the reconstructed dataset, we developed the Noisy Label Filter (NLF) procedure. Specifically, the procedure helps identify additional relevant records that were previously not part of the dataset or missed due to human screening fatigue. The NLF procedure aims to reduce the number of noisy labels by screening a minimal set of records and filtering out any additional relevant records. The core idea of the NLF procedure is to let the active learning model predict the most likely relevant records (based on the initially relevant records) and have a screener label a set of such records. We propose the following steps:

1. Import the reconstructed dataset in screening software implementing active learning.
2. Determine the training set by selecting all relevant records as training data and some random records as irrelevant.
3. Choose the active learning model, including a feature extractor and classifier, and select the query strategy for presenting the most likely relevant record to the screener.
4. Ask an expert to screen a subset of the records using the same inclusion and exclusion criteria used in the initial study and stop after 1–2 h of screening *x*, for example 50 (or more), irrelevant records in a row. After screening many irrelevant records in a row, it is unlikely that any more relevant records will show up, and the time investment should balance between thoroughness in screening and efficiency in the process.

The Noisy Label Filter (NLF) procedure can produce the following results, each with its consequences for the simulation study:

1. All screened records up to the stopping rule are labeled as irrelevant. This outcome means all unseen noisy labels can probably be labeled irrelevant.
2. One or more screened records are labeled as relevant, which can have several explanations, such as:
  - The additional relevant records are articles that were only obtained via the replication search and should have been labeled as relevant if they had been retrieved during the initial search. These records are truly relevant but were not found in the initial database for various reasons, such as updated thesauri or database corrections. Such records can be either removed from the dataset since these were not part of the initial dataset or kept in the data as relevant.
  - The additional relevant records were also found in the initial search but were then falsely labeled as irrelevant due to screening fatigue or human error. Such a noisy label should be re-labeled as relevant unless it is a duplicate; in that case, it can safely be removed.

The output of the NLF procedure is a reconstructed dataset with noisy labels filtered out and all records correctly labeled as relevant or irrelevant (*k*_*r,*+_ or *k*_*r,-*_).

### Application

**Fig. 2:**
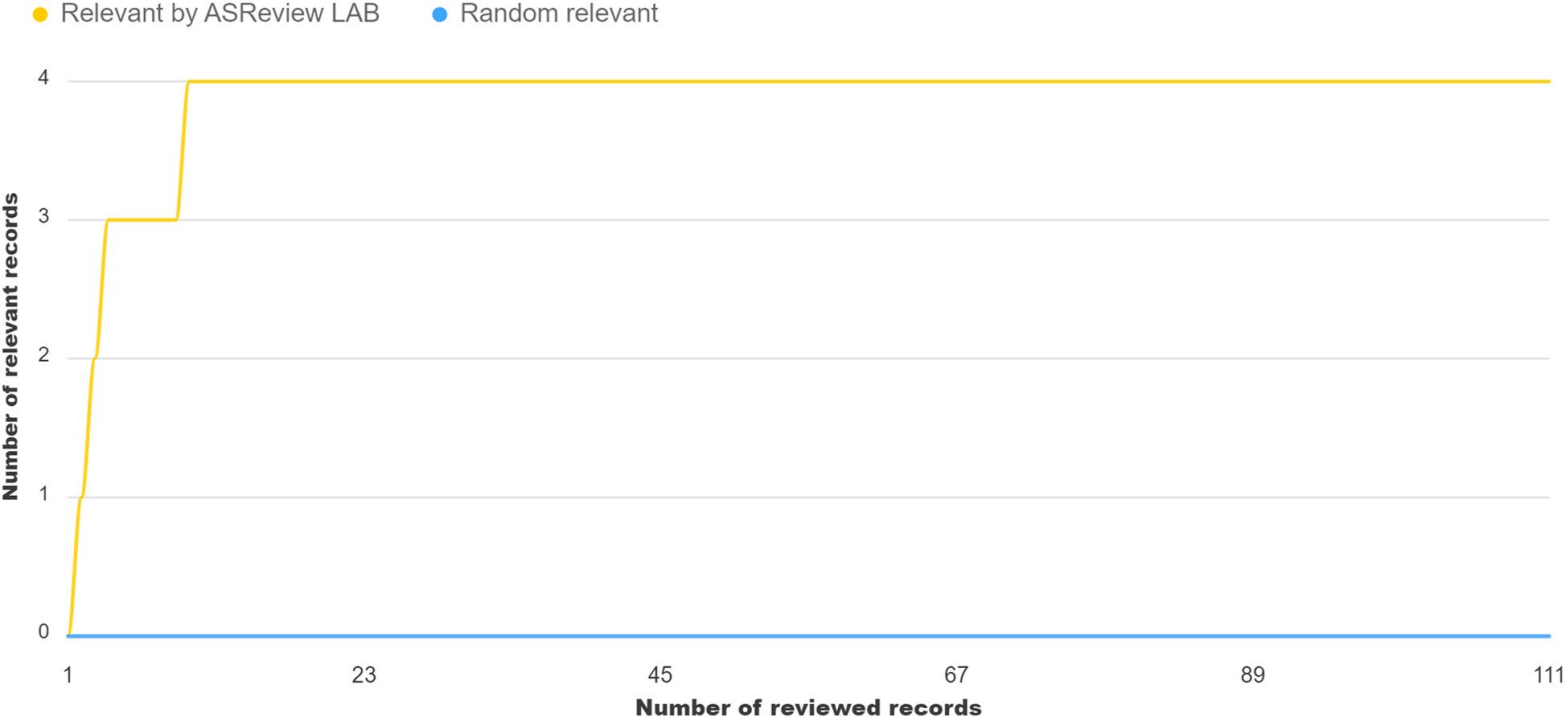
Lapse of screening process in application NLF procedure. The yellow line represents the recall of the four relevant records during the NLF procedure. Notably, the four relevant records were later classified as irrelevant, see Table 2

One of the original researchers, Matthijs Oud (MO), applied the NLF procedure on November 3rd, 2022. We imported the reconstructed dataset in v1.1 of ASReview [2] with the 20 known-relevant records as training data, and we pre-labeled five random records as irrelevant. During the screening phase, which lasted about an hour, the researcher labeled 111 records and stopped after labeling 50 irrelevant records in a row; see Fig. 2 for the recall plot. Four records were labeled as potentially relevant, but after discussion with the team, we labeled the four studies as irrelevant; see Table 2. According to MO, these records were part of the initial dataset and then also excluded. Since our goal was to reproduce this dataset as precisely as possible and since this researcher decided to exclude follow-up papers of an already included paper in his initial paper, these four records were labeled irrelevant.

**Table 2:** Noisy labeled potentially relevant papers

Reference	Description	Final label
[23]	This is a follow-up study of an initially included record [24]	Irrelevant
[38]	This study had an already included follow-up study (Van den [36]	Irrelevant
[20]	The article was a follow-up study of an initially included paper [19]	Irrelevant
Van den [35]	This article seems to be a Dutch translation of an English article and has at least overlapping data (Van den [36]	Irrelevant

Then, we labeled all unseen records as irrelevant, resulting in *k*_*r,-*_ = 1033 records being labeled as such, and the initially relevant studies were kept as relevant, *k*_*r,*+_  = 20, except for the retracted paper [3]. The irrelevant labels were added to the data via ASReview Datatools (De Bruin, 2020/2022). With this labeled dataset, we could proceed to perform a simulation study.

## Simulation study

With the reconstructed dataset, a simulation study was carried out to mimic the screening process, as if the screener had used different active learning models. In the methods section, we describe how this simulation study was conducted. In the results section, we describe the main results of the simulation study.

### Methods

The purpose of the simulation study was to compare the efficiency of the active learning models to manual screening. A simulation study was conducted using ASReview Makita [34]. The ‘multiple models’ template was used to compare Logistic Regression, Naïve Bayes, Random Forest, and Support Vector Machine. The simulation study included one randomly selected relevant record [8] and one randomly selected irrelevant record [16] as prior knowledge. The performance of different active learning models was evaluated by calculating the amount of work that could have been saved using ASReview at a certain level of recall, specifically at 95% recall, known as ‘work saved over sampling’ (WSS@95%) [10], the number of Extra Relevant Records found at 10% recall, and the Average Time to Discover (ATD) a relevant record [14]. In our study, the ATD for each relevant paper is calculated by averaging the Time to Discovery (TD) across all simulation runs for a given active learning model. Then, these averaged TD values are further averaged across all relevant records within a simulation condition. This approach yields a single, representative ATD score for each of the four models we compared, offering a holistic view of their performance.

All outcomes of the Makita script—as well as the script itself—are available on the OSF page [27].

### Results

**Fig. 3:**
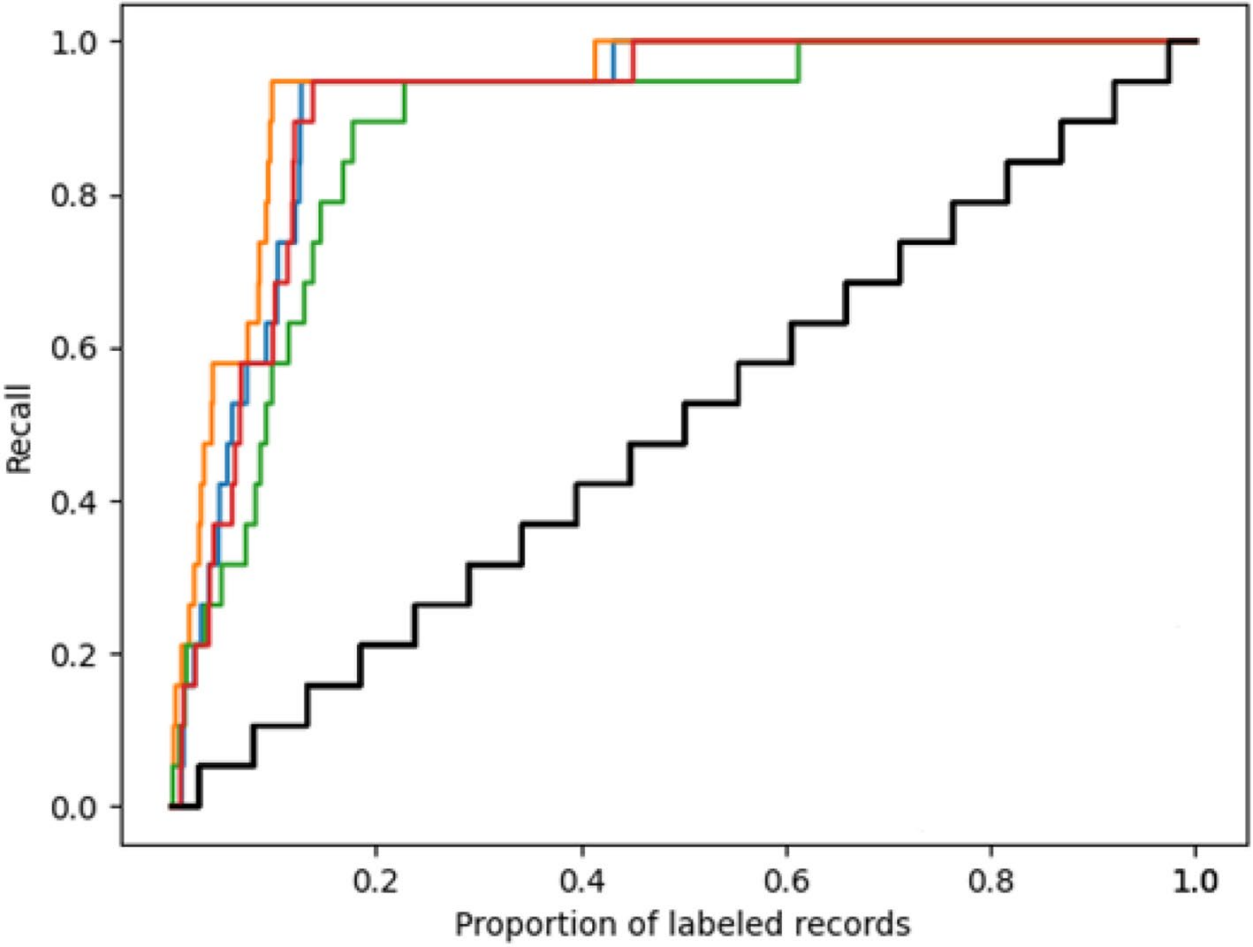
Recall curve of the simulation study. The *x*-axis represents the proportion of labeled records, and the y-axis represents the proportion of relevant records. The black line represents the average process of random screening, while the colored lines represent the performance of different active learning models, with blue for logistic regression, orange for Naive Bayes, green for Random Forest, and red for support vector machine

Figure 3 displays the recall curve, and as can be observed, the Naïve Bayes classifier (orange line) is the first to reach 95% recall, while Random forest (the green line) is the last to reach that level. Remarkably, finding the last relevant record [26] took screening more records than finding any of the other included ones. A possible explanation could be that this article, compared to the others, described relatively similar treatment groups. More specifically, the two experimental groups only differ from each other in allowing patients to call their therapist while otherwise giving them the same intervention [26].

Table 3 shows the WSS@95% for each of the four modes. Naïve Bayes has the highest work saved over sampling at 95% of recall (82,30%), whereas Random Forest has the lowest WSS@95 (69.36%). The other metrics show similar results: After having screened 10% of all records, Naïve Bayes found the most extra relevant records (‘ERF’ = extra relevant records found) compared to manual selection (84.21% ERF) and Random Forest scored lowest again (47.37%). The average time to discovery (ATD) for the relevant records, expressed in the number of records before one relevant record is found, is the lowest for Naïve Bayes (91) and the highest for Random Forest (125). This means that, on average, Naïve Bayes had the least irrelevant records before a relevant record was found, which indicates that Naïve Bayes has the best performance.

**Table 3:** Simulation results

Model	WSS@95	ERF@10	ATD
Logistic Regression	79.44	52.63	91
Naïve Bayes	82.30	84.21	70
Random Forest	69.36	47.36	125
Support Vector Machine	78.30	52.63	93

*WSS * Work saved over sampling, *ERF *Extra relevant records found, *ATD *Average time to discovery

## Discussion

In this study, we explored a method to create a dataset to conduct a simulation study evaluating the performance of active learning models. The information we did have access to consisted of the published search queries and the corresponding number of results, the list of included papers, and the number of screened records. Although the authors of the initial systematic review (i.e., [29]) followed the, at that time, prevailing 2009 and the updated 2020 guidelines [25, 30], the list of excluded records was not available. We discovered that replicating the initial search query 5 years later to reconstruct the initial dataset is only possible to a certain extent, but we could not reproduce the exact same data. There are several explanations, all outside the influence of the original authors, such as search reproduction being limited by differences in institutional access, search strings without specified search fields, and changes in the literature databases over time.

As a result, it remains unknown whether our reproduced dataset contains exactly the initial records. The issue with running a simulation study with such noisy labels is the possibility of additional relevant records: a paper only found by the replication process and relevant according to inclusion criteria. Such a noisy label will hamper the performance of active learning models since a model will be trained with erroneously labeled data. That is why one should not naively label all noisy labels as irrelevant. Therefore, we introduced the Noisy Label Filter (NLF) procedure. In short, a researcher screens the most likely relevant but noisy labels as predicted by a machine-learning model until a pre-specified stopping rule is met. This way, one can test for the presence of additional relevant records with minimum screening effort. The NLF outcome for our case study was that we labeled all noisy labels as irrelevant, with more confidence than without the NLF procedure. Then, we were able to run the simulation study testing the performance of active learning on our reconstructed, fully-labeled dataset.

As this type of research is quite specific and new, there were informational constraints. To the best of our knowledge, there was no other study on reconstructing a database to run a simulation study. Because of that, some decisions in our process and design were made based on personal judgment and intuition rather than prescribed rules and norms. For example, we decided not to include The Cochrane Library database because of viability and expected overlap in records. We believe this did not harm our goal of finding the initial inclusions; all initially included records were retrieved from the other databases. Furthermore, we implemented a provisional stopping rule where screening was halted after approximately one hour or upon labeling 50 consecutive records as irrelevant. The selection of this threshold is subject to ongoing debate within the screening literature. However, we contend that this heuristic likely had minimal impact on our results. This assumption is based on the premise that the records imported into the screening software were predominantly irrelevant, and given that our active learning model prioritizes the presentation of records it classifies as most likely to be relevant, we posited that it was highly unlikely for a new relevant record to emerge after 111 records were screened. We recognize the arbitrariness of this cutoff and acknowledge that the ideal threshold may vary considerably, influenced by the volume of records retrieved from database searches and the complexity inherent to the research question and evidence base. For further discussion and a heuristic for determining a more nuanced stopping rule, we refer readers to the works of [5, 7, 39], and [40] who provide a comprehensive evaluation of this issue. Additionally, see Boetje and van de Schoot [6], for a practical and high-quality heuristic.

There is considerable potential for further research in this area. Our study presents a single case study, and the ease or difficulty of replicating a search could vary substantially across different systematic reviews. Importantly, this case study included the participation of one of the authors from the original systematic review. As one of the reviewers aptly notes, replicating a search without the involvement of the original systematic reviewers could present more challenges in practice. Additionally, it is worth considering that this researcher, while more experienced now, conducted the initial screening several years ago. This gap could have influenced both their perspective and recall of the specific topic, potentially impacting the results of our replication.

Focused on search reproduction, we have concerns regarding the PRISMA guidelines [30]. A key limitation we identified is that while the PRISMA guidelines mandate the reporting of full search strategies for all databases, they lack specificity on what constitutes a ‘full search strategy’. Often, authors limit their reporting to databases used and search terms, omitting critical details such as field codes, access platforms, and institutional access parameters. Reproduction becomes more challenging when thesaurus terms and searches without specific search fields are used, as both differences between access platforms and changes in the thesaurus can affect the retrieved records. These elements are vital for true reproducibility but are frequently overlooked, leading to challenges in replicating systematic reviews accurately. Moreover, the level of description of a “full search strategy” is open to interpretation and reporting of search strategies in systematic reviews is suboptimal (e.g., [4, 18, 21, 22]). So, while these guidelines provide a comprehensive checklist covering various sections of a systematic review manuscript, including the title, abstract, introduction, methods, results, discussion, and other information, they fall short of ensuring complete reproducibility. In theory, adherence to this checklist should guarantee transparency and reproducibility of the systematic review process. However, our findings indicate that this is not always the case in practice. Based on our study, we recommend that the next update of the PRISMA guidelines will comprise more extensive reporting of the search strategies, specifying platforms, institutional access, and field codes besides the search terms, but also storage of the search results and of the full labeled dataset of included and excluded records.

Further research could assess the balance between comprehensiveness and reproducibility. Certain searchable database fields will change more frequently than others; for example, the content and hierarchy behind thesaurus fields will be adapted more frequently than the content of title and abstract terms. Searching thesaurus fields may thus make a reproduction of a search more challenging, but using them is essential for a comprehensive search and, thus, for any systematic review. It might be useful to know which part of the discrepancies was due to each of the individual reasons: differences in institutional access, search strings not specifying search fields, changes in internal dictionaries over time, corrections of errors, retractions, and changes in indexing. With this type of knowledge, the development of initial searches, reporting of searches, and reproduction of searches could all focus on improving reproducibility without losing relevant records. Besides, it would be useful to assess the overlap between search engines. In the current case study, we only know that The Cochrane Library database did not retrieve relevant studies that were not covered by the other databases. It is advantageous to search fewer literature databases for efficiency but also search reproducibility. Again, this is a balancing act with comprehensiveness.

Moreover, it could be useful to conduct other types of simulation studies and compare the outcomes with each other. We decided to classify the relevant records found in the NLF procedure application as irrelevant, meaning we selected all noisy labels as irrelevant. Subsequently, we conducted only one simulation study in ASReview. Then, we compared multiple models and discovered that Naïve Bayes had the best model fit. A relevant topic for further study is to investigate the effect of prior knowledge on the outcomes of simulation studies. In ASReview Makita, this is possible using the ARFI template De Bruin [11, 12]. In this template, the effect of changing relevant records as prior knowledge could be investigated relatively easily, keeping irrelevant records as prior knowledge constant. If the outcome of particular simulation studies with specific relevant records as prior knowledge differs significantly from the other relevant records’ simulation studies, we could investigate why this difference exists and how these particular relevant records differ from the other relevant records. With this comparison, we could increase our understanding of the effect of prior knowledge of relevant records on simulation studies, ultimately improving the machine learning-aided screening process.

Furthermore, besides additional tests of the NLF procedure in other case studies, it may be helpful to investigate whether the NLF procedure can be used for different purposes. In short, the NLF procedure filters all noisy labels (in our case, all non-inclusions) into potentially relevant and irrelevant records. In our study, this was done to ensure we did not miss any additional relevant records that could be found using the reconstructed queries. However, this NLF procedure could potentially be used as a second check if the screening process did not miss any relevant records. Further research could, for example, focus on the question of how to double-check the initial screening process by using the NLF procedure for a single original review. And if it yields the discovery of one or more falsely-labeled records, it is relevant to investigate the effect of adding/deleting these records to the meta-analysis. Last, further research with additional case studies could result in a general guide on reproducing searches and conducting simulation studies without access to fully-labeled datasets. Our first thoughts are described in the next section.

### A decision tree

**Fig. 4:**
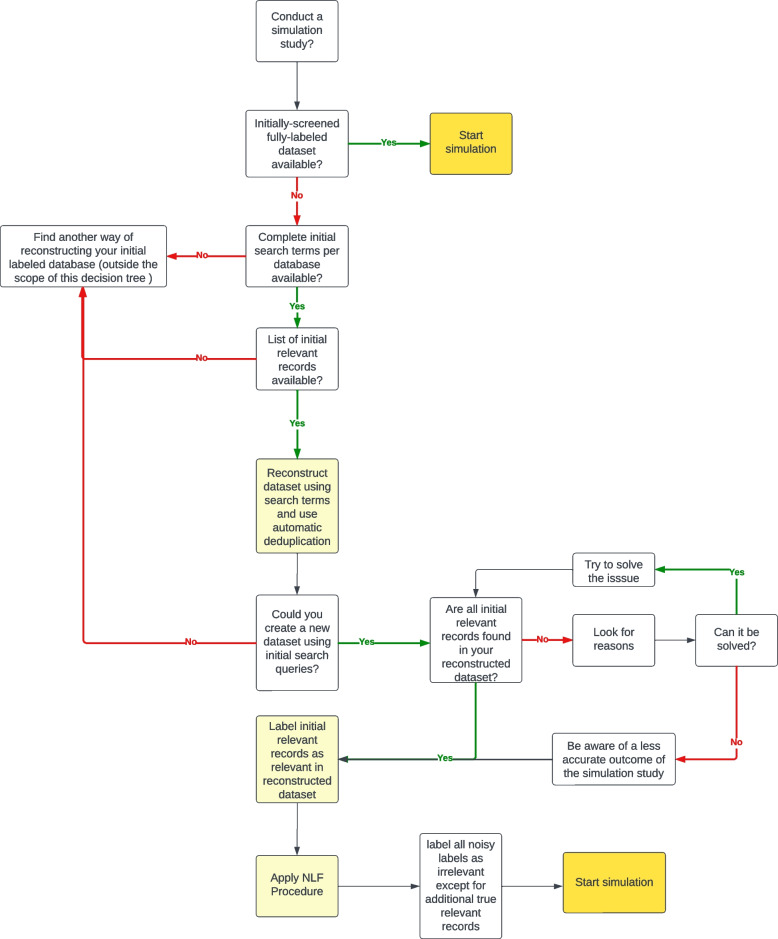
Simulation decision tree

Lastly, we introduce a Decision Tree in Fig. 4, which you can use to undertake a simulation study to assess the efficacy of active learning models for a labeled systematic review dataset. A simulation study necessitates an initially screened dataset, incorporating the titles and abstracts derived from the search and the labeling decision that emerges from the screening process. If such a dataset is readily accessible, you may commence the simulation study immediately. However, if the initially screened dataset is unavailable, reconstruction of the dataset using search queries, as demonstrated in our current study, is required. The prerequisites for employing this decision tree are as follows:

- 1. The full set of initial search terms per database must be obtainable;
- 2. The list of initially relevant records must be on hand;
- 3. Information regarding the number of results retrieved per database for each search query must be accessible;

Once these prerequisites are met, one is well-positioned to commence the process of dataset reconstruction. This involves a systematic approach, meticulously following the steps detailed in the decision tree.

The first stage of this process is preparation, which involves gathering all necessary materials and ensuring that they are in the appropriate format for the reconstruction. This primarily includes obtaining the initial search terms for each database, the list of initially relevant records, and the number of results retrieved per database for each search query. The second stage is the actual reconstruction, where the data is recompiled based on the provided search queries. This step requires careful attention to detail and adherence to the guidelines outlined in the decision tree. Here, the importance of the initial search terms and the list of initially relevant records becomes evident as they serve as the foundation for the reconstructed dataset. The final stage involves the verification and validation of the reconstructed dataset. At this point, the reconstructed dataset is compared against the list of initially relevant records to ensure accuracy. Any discrepancies need to be identified and corrected to ensure the dataset’s integrity. The decision tree results in a dataset that accurately represents the initial dataset, enabling running simulation studies. We recommend caution: if the numbers differ by more than 5% from the original publication or if search queries generate errors, the reconstruction of the initial database might pose substantial risks.

Despite the inherent complexities and challenges, our decision tree for reconstructing systematic review datasets offers an exciting prospect for advancing active learning in this critical field. It paves the way for reducing the labor intensity of systematic reviews, thereby potentially accelerating the production of high-quality research. As we continue to refine and evolve this methodology with further studies and use cases, we anticipate that our contributions will significantly enhance the efficiency and efficacy of future systematic reviews.

### Generative AI usage

During the preparation of this work, the authors used Grammarly to improve the language and readability but did not generate any IP with these tools. After using these tools, the authors reviewed and edited the content as needed and take full responsibility for the content of the publication.

### Open science statements: data, and code availability

The datasets supporting the conclusions of this article are available in the Open Science Framework repository [27], 10.17605/OSF.IO/PJR97.

1. Reconstruction
  In the document’ *reconstructed_data_after_reproducting_search_queries.ris*’, the list of records after exporting but before deduplication can be found. In other words, these are the data after exporting the relevant search queries, without having performed any correction yet.
  In the document ‘reconstructed_data_after_quality_check_2.ris’, the reconstructed data have been adjusted following the steps’ quality check 1, deduplication, and quality check 2’. This means the data are deduplicated, and the initially relevant records are labeled so (‘ASReview_relevant’) and all the other records – the noisy labels – are labeled as ‘ASReview_not_seen’ (by the *ASReview datatools* script referred to in the paper.)
2. Applying NLF procedure
  The document’ nlf_procedure_test_trimbos.asreview’ can be opened in ASReview. The NLF procedure was performed in ASReview, and the ASReview file was exported.
  In the document ‘reconstructed_data_after_NLF_procedure.ris’, following the results of the NLF procedure, all noisy labels were labeled as irrelevant. Now the relevant records are labeled as ‘ASReview_relevant’ and the irrelevant records are labeled as ‘ASReview_irrelevant’.
3. Simulation
  In the folder ‘Simulation_Makita’, all relevant data concerning the simulation study can be found. For example, one can see the list of records used, the ASReview statistics, and a graph in which different simulation study modes can be compared.

### Supplementary Information

**Additional file 1: Appendix 1.** Overview of exported queries. **Table A.1.** Field codes and search syntax per platform. **Table A.2.** PsycInfo. **Table A.3.** PubMed. **Table A.4.** CinaHL. **Table A.5.** Embase. **Appendix 2.** Deduplication.
