# Supplementary material for: Addressing the challenges of reconstructing systematic reviews datasets: a case study and a noisy label filter procedure
Source: Syst Rev. 2024 Feb 17;13:69. doi: 10.1186/s13643-024-02472-w (PMC10874047; doi:10.1186/s13643-024-02472-w)
Supplement: Supplementary file 1 — Additional file 1: Appendix 1. Overview of exported queries. Table A.1. Field codes and search syntax per platform. Table A.2. PsycInfo. Table A.3. PubMed. Table A.4. CinaHL. Table A.5. Embase. Appendix 2. Deduplication. [file 13643_2024_2472_MOESM1_ESM.docx]

Appendices

Appendix A provides the syntax for the initial and reconstructed queries, with the number of papers found for each sub-query. In Appendix B, we describe the deduplication phases in detail.

### Appendix A: Overview of exported queries

#### At the time of the search reconstruction, Utrecht University Library subscribed to the same databases but via different platforms as compared to the initial researchers. Therefore, the initial search queries could not be repeated on precisely the same platforms and had to be translated to the other platforms. Table A.1 lists the changes made to the syntax and the expected impact this had on the results. The aim was to replicate the initial searches as closely as possible without checking all individual thesaurus terms and field codes. When there were several options, the most sensitive option was always chosen to prevent missing relevant records in the results. The last column indicates the expected impact. No impact means the search should retrieve the same results. However, the search reconstruction was performed five years after the initial search. Because of changes in the databases since the original search, we did not expect to retrieve the same number of results even with a date filter.

Tables A.2-A.5 provide field codes from the original and replicated search, with the numbers of results. Due to different syntax, an identical search looks different when run on a different platform. The tables contain the original searches and the reconstructed searches in the same database, but often different platforms. When the changes in search query are not because of differing syntax or field codes, this is explained in the last column “*Changes in queries”*. Of note: A field code refers to the way specific fields are noted (e.g., “ti.” as title). With a search term, we mean the subject we are looking for within a field (e.g., “Psychotherapy). The syntax refers to the way different field codes and search terms are combined in the search.

Table A.1 Field codes and search syntax per platform

|  | **Psychinfo** | | **PubMed** | | **Embase** | | **Expected impact on results** |
| --- | --- | --- | --- | --- | --- | --- | --- |
| **Search fields** | *Original search –probably EBSCOhost (2017)* | *Reconstructed search – Ovid (2022)* | *Original search – probably Pubmed.gov Legacy*  *(2017)* | *Reconstructed search –Pubmed.gov**  *Current version*  *(2022)* | *Original search –*  *Probably Ovid*  *(2017)* | *Reconstructed searches –*  *Embase.com (2022)* |  |
| *Title* | TI | ti. | [Title] | [Title] | .ti. | ti | None |
| *Abstract* | AB | ab. | [Abstract]* | [Abstract]* | .ab. | ab | None |
| *Author keywords* | KW | Id. | * Included in Abstract field | * Included in Abstract field | (.kw. not used) | kw | Low |
| *Subject Heading* | DE | / | [Mesh:NoExp] | [Mesh:NoExp] | / | /de | None (although the use of the subject heading may have changed over time) |
| *Subject Heading – explode* | Exp | Exp | [MeSH] | [MeSH] | Exp | /exp | High; the explode function only works one level down via EBSCOhost, while the entire hierarchy is included via the other platforms. |
| *Classification code* | CC | .cc | n.a. | n.a. | n.a. | n.a. | None (although the use of the classification code may have changed over time) |
| *Subset* | n.a. | n.a. | [sb]** | [sb]** | n.a. | n.a. | Low |
| *Proximity* | NEAR/*n* (within *n* words) | Adj*k* (k=n+1; within *n* words | n.a. | n.a. | ? | NEAR/n within n words | None to Low |
| *Date Limit* | ? | limits | ? | limits | ? | limits | Low (update frequency and dates can vary) |
| *Study and Publication types* | limits | limits | systematic[sb]  [pt] | manually adapted**  [pt] | Aspecific search | Subject heading | Uncertain |

*n.a.:* not applicable. Cinahl is not listed in the table as both the original and the reconstructed search were performed via EBSCOhost.

?: unknown

*The Pubmed platform underwent a substantial change between the original and the reconstructed search dates.

**The Medline filter changed between the original and the reconstructed search date. Thus, the filter was replaced by a manual search string corresponding to the old version of the filter for PubMed.

Table A.2**: PsycInfo.**

|  | | **#results (June 2017)** | **Query New (Ovid)** | | **#results**  **(1-11-2022)^[[1]](#footnote-2)^** | **Changes in queries** |
| --- | --- | --- | --- | --- | --- | --- |
| S15 | **S11 (study type: systematic review or meta analysis)** | **55** | S15 | ***limit 15 to up=19000101-20170630*** | **41** | Limited to date range of initial search |
|  |  |  |  | limit 11 to ("0830 systematic review" or 1200 meta analysis) | 92 | Adjusted field code and syntax, identical search terms. |
| S14 | S11 | 5,917 | S14 | S11 | 6,067 | Fully identical |
| S13 | **S11 AND S12** | **478** | S13 | ***limit 13 to up=19000101-20170630*** | ***524*** | Limited to date range of initial search |
|  |  |  |  | S11 AND S12 | 773 | Fully identical |
| S12 | placebo* OR random* OR "comparative stud*" OR (clinical NEAR/3 trial*) OR (research NEAR/3 design) OR (evaluat* NEAR/3 stud*) OR (prospectiv* NEAR/3 stud*) OR ((singl* OR doubl* OR trebl* OR tripl*) NEAR/3 (blind* OR mask*)) | 185,675 | S12 | (placebo* or random* or "comparative stud*" or (clinical adj4 trial*) or (research adj4 design) or (evaluat* adj4 stud*) or (prospectiv* adj4 stud*) or ((singl* or doubl* or trebl* or tripl*) adj4 (blind* or mask*))).ab,ti. | 432,087 | Adjusted field codes and syntax, identical search terms.  Near/3 changed into adj4: both hit with a maximum of 3 words between the intented search terms. In the initial query, there were no field codes specified, and we are not sure which fields were searched with or without limitations. Selecting all fields in the reconstructed query, led to even more results than the current 432,087; so we pragmatically restricted to the most relevant fields: abstract and title. |
| S11 | S3 AND S9 (publication type: all jounals) | 5,917 | S11 | limit 10 to all journals | 6,067 | Adjusted field code and syntax, identical search term.  ‘ |
| S10 | S3 AND S9 | 7,753 | S10 | S3 AND S9 | 8,226 | Fully identical |
| S9 | S7 OR S8 | 943,985 | S9 | S7 OR S8 | 960,607 | Fully identical |
| S8 | CC 3300 OR 331* OR 335* OR 337* OR 338* OR 3365 | 877,750 | S8 | ("3300" or 331* or 335* or 337* or 338* or "3365").cc. | 890,356 | Adjusted field codes and syntax, identical search terms. |
| S7 | S4 OR S5 OR S6 | 277,569 | S7 | S4 OR S5 OR S6 | 302,877 | Fully identical |
| S6 | TI ( ( "Transference Focused Psychotherapy" OR "Schema-Focused Therapy" OR "Dialectical Behavior Therapy" OR "Mentalisation Based Treatment" OR "Mentalization Based Treatment" OR psychotherapy ) OR KW ( ( "Transference Focused Psychotherapy" OR "Schema-Focused Therapy" OR "Dialectical Behavior Therapy" OR "Mentalisation Based Treatment" OR "Mentalization Based Treatment" OR psychotherapy ) | 66,052 | S6 | ("Transference Focused Psychotherapy" or "Schema-Focused Therapy" or "Dialectical Behavior Therapy" or "Mentalisation Based Treatment" or "Mentalization Based Treatment" or psychotherapy).id,ti. | 76,390 | Adjusted field codes and syntax, identical search terms. ‘KW’ [keyword] changed into ‘id’ [key concept], which mimics EBSCO’s ‘keyword’ the best. |
| S5 | DE "Dialectical Behavior Therapy" | 932 | S5 | dialectical behavior therapy/ | 1,743 | Adjusted field codes and syntax. |
| S4 | DE "Psychotherapy" OR DE "Adlerian Psychotherapy" OR DE "Adolescent Psychotherapy" OR DE "Analytical Psychotherapy" OR DE "Autogenic Training" OR DE "Behavior Therapy" OR DE "Brief Psychotherapy" OR DE "Brief Relational Therapy" OR DE "Child Psychotherapy" OR DE "Client Centered Therapy" OR DE "Cognitive Behavior Therapy" OR DE "Conversion Therapy" OR DE "Eclectic Psychotherapy" OR DE "Emotion Focused Therapy" OR DE "Existential Therapy" OR DE "Experiential Psychotherapy" OR DE "Expressive Psychotherapy" OR DE "Eye Movement Desensitization Therapy" OR DE "Feminist Therapy" OR DE "Geriatric Psychotherapy" OR DE "Gestalt Therapy" OR DE "Group Psychotherapy" OR DE "Guided Imagery" OR DE "Humanistic Psychotherapy" OR DE "Hypnotherapy" OR DE "Individual Psychotherapy" OR DE "Insight Therapy" OR DE "Integrative Psychotherapy" OR DE "Interpersonal Psychotherapy" OR DE "Logotherapy" OR DE "Narrative Therapy" OR DE "Network Therapy" OR DE "Persuasion Therapy" OR DE "Primal Therapy" OR DE "Psychoanalysis" OR DE "Psychodrama" OR DE "Psychodynamic Psychotherapy" OR DE "Psychotherapeutic Counseling" OR DE "Rational Emotive Behavior Therapy" OR DE "Reality Therapy" OR DE "Relationship Therapy" OR DE "Solution Focused Therapy" OR DE "Supportive Psychotherapy" OR DE "Transactional Analysis" OR DE "Behavior Therapy" OR DE "Aversion Therapy" OR DE "Conversion Therapy" OR DE "Dialectical Behavior Therapy" OR DE "Exposure Therapy" OR DE "Implosive Therapy" OR DE "Reciprocal Inhibition Therapy" OR DE "Response Cost" OR DE "Systematic Desensitization Therapy" OR DE "Cognitive Behavior Therapy" OR DE "Acceptance and Commitment Therapy" OR DE "Gestalt Therapy" OR DE "Empty Chair Technique" OR DE "Group Psychotherapy" OR DE "Encounter Group Therapy" OR DE "Therapeutic Community" OR DE "Humanistic Psychotherapy" OR DE "Client Centered Therapy" OR DE "Hypnotherapy" OR DE "Age Regression (Hypnotic)" OR DE "Ericksonian Psychotherapy" OR DE "Psychoanalysis" OR DE "Adlerian Psychotherapy" OR DE "Brief Relational Therapy" OR DE "Dream Analysis" OR DE "Self Analysis" OR DE "Psychotherapeutic Counseling" OR DE "Family Therapy" OR DE "Cognitive Therapy" OR DE "Cotherapy" OR DE "Couples Therapy" OR DE "Educational Therapy" OR DE "Holistic Health" OR DE "Online Therapy" OR DE "Paradoxical Techniques" OR DE "Pastoral Counseling" OR DE "Phototherapy" OR DE "Psychotherapeutic Processes" OR DE "Countertransference" OR DE "Insight (Psychotherapeutic Process)" OR DE "Negative Therapeutic Reaction" OR DE "Psychotherapeutic Breakthrough" OR DE "Psychotherapeutic Resistance" OR DE "Psychotherapeutic Transference" OR DE "Therapeutic Alliance" OR DE "Psychotherapeutic Techniques" OR DE "Active Listening" OR DE "Animal Assisted Therapy" OR DE "Autogenic Training" OR DE "Brief Relational Therapy" OR DE "Cotherapy" OR DE "Dream Analysis" OR DE "Empty Chair Technique" OR DE "Ericksonian Psychotherapy" OR DE "Guided Imagery" OR DE "Mirroring" OR DE "Morita Therapy" OR DE "Motivational Interviewing" OR DE "Mutual Storytelling Technique" OR DE "Network Therapy" OR DE "Paradoxical Techniques" OR DE "Psychodrama" OR DE "Recreation Therapy" OR DE "Spontaneous Remission" OR DE "Theoretical Orientation" | 270,571 | S4 | exp Psychotherapy/ or exp Behavior Therapy/ or cognitive behavior therapy/ or "acceptance and commitment therapy"/ or exp group psychotherapy/ or exp Hypnotherapy/ or exp psychoanalysis/ or family therapy/ or cognitive therapy/ or educational therapy/ or holistic health/ or online therapy/ or pastoral counseling/ or phototherapy/ or exp psychotherapeutic processes/ or exp psychotherapeutic techniques/ or recreation therapy/ or spontaneous remission/ or theoretical orientation/ | 293,296 | Adjusted field codes, syntax and search terms.  This query changed into a smaller, more readable one. Many concepts in the initial query were subconcepts of exploded concepts in the new query. The initial concepts that were not covered by exploded concepts in the new queries, were also included in the new query independently. That is why some concepts were assigned ‘exp’ (explode) in the reconstructed query, which covered many subconcepts in the initial query, and other concepts were not exploded (‘concept’/). However, some subconcepts that were covered by the reconstructed query, were not covered by the initial query, e.g., affirmative therapy, strategic therapy, posthypnotic suggestions, contemporaneity, enactments, psychotherapeutic neutrality, centering, free association, and life review. |
| S3 | S1 OR S2 | 18,861 | S3 | S1 OR S2 | 22,499 | Fully identical |
| S2 | TI borderline OR AB borderline OR KW borderline | 18,256 | S2 | borderline.ab,id,ti. | 22,324 | Adjusted field codes and syntax, identical search terms.  Field code ‘KW’ [keyword] changed into ‘id’ [key concept], which mimics EBSCO’s ‘keyword’ the best. |
| S1 | (DE "Borderline Personality Disorder") OR (DE "Borderline States") | 10,336 | S1 | borderline personality disorder/ or borderline states/ | 11,686 | Adjusted field codes and syntax, identical search terms. |

Table A.3: **Pubmed**

| **Query old  (probably Pubmed)** | | **#results (June 2017)** | **Query New  (Pubmed)** | | **#results (18-10-2022)** | **Changes in queries** |
| --- | --- | --- | --- | --- | --- | --- |
| [#17](https://www.ncbi.nlm.nih.gov/pubmed/advanced) | Search systematic[sb] AND (#11) | **156** | [#1](https://www.ncbi.nlm.nih.gov/pubmed/advanced)3 | #13 AND ("1900/01/01"[Date - Entrez] : "2017/06/30"[Date - Entrez]) | **177** | Limited to date range of initial search |
|  |  |  |  | ((systematic review [ti] OR meta-analysis [pt] OR meta-analysis [ti] OR systematic literature review [ti] OR this systematic review [tw] OR pooling project [tw] OR (systematic review [tiab] AND review [pt]) OR meta synthesis [ti] OR meta-analy*[ti] OR integrative review [tw] OR integrative research review [tw] OR rapid review [tw] OR umbrella review [tw] OR consensus development conference [pt] OR practice guideline [pt] OR drug class reviews [ti] OR cochrane database syst rev [ta] OR acp journal club [ta] OR health technol assess [ta] OR evid rep technol assess summ [ta] OR jbi database system rev implement rep [ta]) OR (clinical guideline [tw] AND management [tw]) OR ((evidence based[ti] OR evidence-based medicine [mh] OR best practice* [ti] OR evidence synthesis [tiab]) AND (review [pt] OR diseases category[mh] OR behavior and behavior mechanisms [mh] OR therapeutics [mh] OR evaluation study[pt] OR validation study[pt] OR guideline [pt] OR pmcbook)) OR ((systematic [tw] OR systematically [tw] OR critical [tiab] OR (study selection [tw]) OR (predetermined [tw] OR inclusion [tw] AND criteri* [tw]) OR exclusion criteri* [tw] OR main outcome measures [tw] OR standard of care [tw] OR standards of care [tw]) AND (survey [tiab] OR surveys [tiab] OR overview* [tw] OR review [tiab] OR reviews [tiab] OR search* [tw] OR handsearch [tw] OR analysis [ti] OR critique [tiab] OR appraisal [tw] OR (reduction [tw]AND (risk [mh] OR risk [tw]) AND (death OR recurrence))) AND (literature [tiab] OR articles [tiab] OR publications [tiab] OR publication [tiab] OR bibliography [tiab] OR bibliographies [tiab] OR published [tiab] OR pooled data [tw] OR unpublished [tw] OR citation [tw] OR citations [tw] OR database [tiab] OR internet [tiab] OR textbooks [tiab] OR references [tw] OR scales [tw] OR papers [tw] OR datasets [tw] OR trials [tiab] OR meta-analy* [tw] OR (clinical [tiab] AND study [tiab]) OR treatment outcome [mh] OR treatment outcome [tw] OR pmcbook)) NOT (letter [pt] OR newspaper article [pt])) AND #11 | 281 | Identical field codes with adjusted underlyig search scope, identical syntax, identical search terms..  Field code ‘systematic [sb]’ changed in its underlying search scope over time (National Library of Medicine, 2019). We decided to choose the underlying search scope that was the current one in June 2017 (Internet archive (archive.org), 2017). That is why we chose to use the complete underlying search scope of june 2017 + ‘AND #11’. |
| [#15](https://www.ncbi.nlm.nih.gov/pubmed/advanced) | Search (Therapy/Narrow[filter]) AND (#11) | [**288**](https://www.ncbi.nlm.nih.gov/pubmed/?cmd=HistorySearch&querykey=15) | [#1](https://www.ncbi.nlm.nih.gov/pubmed/advanced)2 | #12 AND ("1900/01/01"[Date - Entrez] : "2017/06/30"[Date - Entrez]) | **309** | Limited to date range of initial search |
|  |  |  |  | (Therapy/Narrow[filter]) AND (#11) | 449 | Fully identical |
| [#11](https://www.ncbi.nlm.nih.gov/pubmed/advanced) | Search (#5 OR #10) | [4971](https://www.ncbi.nlm.nih.gov/pubmed/?cmd=HistorySearch&querykey=11) | [#11](https://www.ncbi.nlm.nih.gov/pubmed/advanced) | #5 or #10 | 6379 | Fully identical |
| [#10](https://www.ncbi.nlm.nih.gov/pubmed/advanced) | Search (#4 AND #9) | [1960](https://www.ncbi.nlm.nih.gov/pubmed/?cmd=HistorySearch&querykey=10) | [#10](https://www.ncbi.nlm.nih.gov/pubmed/advanced) | #4 and #9 | 2556 | Fuly identical |
| [#9](https://www.ncbi.nlm.nih.gov/pubmed/advanced) | Search (#6 OR #7 OR #8) | [174083](https://www.ncbi.nlm.nih.gov/pubmed/?cmd=HistorySearch&querykey=9) | [#9](https://www.ncbi.nlm.nih.gov/pubmed/advanced) | #6 or #7 or #8 | 215887 | Fully identical |
| [#8](https://www.ncbi.nlm.nih.gov/pubmed/advanced) | Search "psychological therapies" [tiab] OR "psychological therapy" [tiab] | [1513](https://www.ncbi.nlm.nih.gov/pubmed/?cmd=HistorySearch&querykey=8) | [#8](https://www.ncbi.nlm.nih.gov/pubmed/advanced) | "psychological therapies" [tiab] OR "psychological therapy" [tiab] | 2710 | Fully identical |
| [#7](https://www.ncbi.nlm.nih.gov/pubmed/advanced) | Search "Dialectical Behavior Therapy" [tiab] OR "Transference Focused Psychotherapy" [tiab] OR "Schema-Focused Therapy" [tiab] OR "Mentalisation Based Treatment" [tiab] OR "Mentalization Based Treatment" [tiab] | [509](https://www.ncbi.nlm.nih.gov/pubmed/?cmd=HistorySearch&querykey=7) | [#7](https://www.ncbi.nlm.nih.gov/pubmed/advanced) | "Dialectical Behavior Therapy" [tiab] OR "Transference Focused Psychotherapy" [tiab] OR "Schema-Focused Therapy" [tiab] OR "Mentalisation Based Treatment" [tiab] OR "Mentalization Based Treatment" [tiab] | 972 | Fully identical |
| [#6](https://www.ncbi.nlm.nih.gov/pubmed/advanced) | Search "Psychotherapy"[Mesh] | [173230](https://www.ncbi.nlm.nih.gov/pubmed/?cmd=HistorySearch&querykey=6) | [#6](https://www.ncbi.nlm.nih.gov/pubmed/advanced) | "Psychotherapy"[Mesh] | 214242 | Fully identical |
| [#5](https://www.ncbi.nlm.nih.gov/pubmed/advanced) | Search ("Borderline Personality Disorder/prevention and control"[Mesh] OR "Borderline Personality Disorder/psychology"[Mesh] OR "Borderline Personality Disorder/rehabilitation"[Mesh] OR "Borderline Personality Disorder/therapy"[Mesh]) | [4548](https://www.ncbi.nlm.nih.gov/pubmed/?cmd=HistorySearch&querykey=5) | [#5](https://www.ncbi.nlm.nih.gov/pubmed/advanced) | ("Borderline Personality Disorder/prevention and control"[Mesh] OR "Borderline Personality Disorder/psychology"[Mesh] OR "Borderline Personality Disorder/rehabilitation"[Mesh] OR "Borderline Personality Disorder/therapy"[Mesh]) | 5785 | Fully identical |
| [#4](https://www.ncbi.nlm.nih.gov/pubmed/advanced) | Search (#1 OR #3) | [10196](https://www.ncbi.nlm.nih.gov/pubmed/?cmd=HistorySearch&querykey=4) | [#4](https://www.ncbi.nlm.nih.gov/pubmed/advanced) | #1 or #3 | 13734 | Fully identical |
| [#3](https://www.ncbi.nlm.nih.gov/pubmed/advanced) | Search borderline [tiab] AND (disorder* [tiab] OR personality [tiab]) | [8780](https://www.ncbi.nlm.nih.gov/pubmed/?cmd=HistorySearch&querykey=3) | [#3](https://www.ncbi.nlm.nih.gov/pubmed/advanced) | borderline [tiab] AND (disorder* [tiab] OR personality [tiab]) | 12225 | Fully identical |
| [#2](https://www.ncbi.nlm.nih.gov/pubmed/advanced) | Search borderline [tiab] AND (disorder [tiab] OR personality [tiab]) | [7802](https://www.ncbi.nlm.nih.gov/pubmed/?cmd=HistorySearch&querykey=2) | [#2](https://www.ncbi.nlm.nih.gov/pubmed/advanced) | borderline [tiab] AND (disorder [tiab] OR personality [tiab]) | 10957 | Fully identical |
| [#1](https://www.ncbi.nlm.nih.gov/pubmed/advanced) | Search "Borderline Personality Disorder"[Mesh] | [5762](https://www.ncbi.nlm.nih.gov/pubmed/?cmd=HistorySearch&querykey=1) | [#1](https://www.ncbi.nlm.nih.gov/pubmed/advanced) | "Borderline Personality Disorder"[Mesh] | 7783 | Fully identical |

Table A.4: **CinaHL**

| **Query old (probably EBSCO)** | | **#results (June 2017)** | **Query New  (EBSCO)** | | **#restults (7-11-2022)** | **Changes in queries** |
| --- | --- | --- | --- | --- | --- | --- |
| S13 | S9  (publ type: meta-analysis, systematic review) | **20** | S13 | S13 and EM 19000101-20170630 | **35** | Limited to date range of initial search |
|  |  |  |  | S9 (publ type: meta-analysis, systematic review) | 75 | Fully identical |
| S12 | S9 | 787 | S12 | S9 | 1,725 | Fully identical |
| S11 | S9 AND S10 | **96** | S11 | S11 and EM 19000101-20170630 | **166** | Limited to date range of initial search |
|  |  |  |  | S9 AND S10 | 326 | Fully identical |
| S10 | placebo* OR random* OR “comparative stud*” OR (clinical NEAR/3 trial*) OR (research NEAR/3 design) OR (evaluat* NEAR/3 stud*) OR (prospectiv* NEAR/3 stud*) OR ((singl* OR doubl* OR trebl* OR triply*) NEAR/3 (blind* OR mask*)) | 274,020 | S10 | placebo* OR random* OR “comparative stud*” OR (clinical NEAR/3 trial*) OR (research NEAR/3 design) OR (evaluat* NEAR/3 stud*) OR (prospectiv* NEAR/3 stud*) OR ((singl* OR doubl* OR trebl* OR triply*) NEAR/3 (blind* OR mask*)) | 870,549 | Fully identical |
| S9 | S7 OR S8 | 787 | S9 | S7 OR S8 | 1,725 | Fully identical |
| S8 | (MH “Borderline Personality Disorder/TH/RH/PC/NU”) | 510 | S8 | (MH “Borderline Personality Disorder/TH/RH/PC/NU”) | 1,027 | Fully identical |
| S7 | S5 AND S6 | 617 | S7 | S5 AND S6 | 1,414 | Fully identical |
| S6 | S1 OR S2 | 4,379 | S6 | S1 OR S2 | 11,939 | Fully identical |
| S5 | S3 OR S4 | 109,357 | S5 | S3 OR S4 | 236,766 | Fully identical |
| S4 | TI ( ( “Transference Focused Psychotherapy” OR “Schema-Focused Therapy” OR “Dialectical Behavior Therapy” OR “Mentalisation Based Treatment” OR “Mentalization Based Treatment” OR psychotherapy OR psychotherapeutic* OR psychotherapist* ) ) OR AB ( ( “Transference Focused Psychotherapy” OR “Schema-Focused Therapy” OR “Dialectical Behavior Therapy” OR “Mentalisation Based Treatment” OR “Mentalization Based Treatment” OR psychotherapy OR psychotherapeutic* OR psychotherapist* ) ) | 6,263 | S4 | TI ( ( “Transference Focused Psychotherapy” OR “Schema-Focused Therapy” OR “Dialectical Behavior Therapy” OR “Mentalisation Based Treatment” OR “Mentalization Based Treatment” OR psychotherapy OR psychotherapeutic* OR psychotherapist* ) ) OR AB ( ( “Transference Focused Psychotherapy” OR “Schema-Focused Therapy” OR “Dialectical Behavior Therapy” OR “Mentalisation Based Treatment” OR “Mentalization Based Treatment” OR psychotherapy OR psychotherapeutic* OR psychotherapist* ) ) | 18,937 | Fully identical |
| S3 | (MH “Psychotherapy+”) OR (MH “Behavior Modification+”) OR (MH “Hypnosis+”) OR (MH “Psychotherapeutic Processes+”) OR (MH “Socioenvironmental Therapy+”) OR (MH “Support, Psychosocial+”) OR (MH “Rehabilitation, Psychosocial+”) OR (MH “Transference (Psychology)+”) OR (MH “Behavior Therapy+”) OR (MH “Cognitive Therapy+”) OR (MH “Desensitization, Psychologic+”) OR (MH “Relaxation Techniques+”) OR (MH “Psychotherapy, Group+”) OR (MH “Psychodrama+”) OR (MH “Symbolism (Psychology)+”) | 106,871 | S3 | (MH “Psychotherapy+”) OR (MH “Behavior Modification+”) OR (MH “Hypnosis+”) OR (MH “Psychotherapeutic Processes+”) OR (MH “Socioenvironmental Therapy+”) OR (MH “Support, Psychosocial+”) OR (MH “Rehabilitation, Psychosocial+”) OR (MH “Transference (Psychology)+”) OR (MH “Behavior Therapy+”) OR (MH “Cognitive Therapy+”) OR (MH “Desensitization, Psychologic+”) OR (MH “Relaxation Techniques+”) OR (MH “Psychotherapy, Group+”) OR (MH “Psychodrama+”) OR (MH “Symbolism (Psychology)+”) | 228,842 | Fully identical |
| S2 | TI borderline OR AB borderline | 4,088 | S2 | TI borderline OR AB borderline | 11,380 | Fully identical |
| S1 | MH “Borderline Personality Disorder” | 1,464 | S1 | MH “Borderline personality disorder” | 3,483 | Fully identical |

Table A.5: **Embase**

| **Query old (probably Ovid)** | | **#results (June 2017)** | **Query New  (embase.com)** | | **#restults (13-10-2022)** | **Changes in queries** |
| --- | --- | --- | --- | --- | --- | --- |
| 1 | exp borderline state/ | 11185 | 1 | ‘borderline state’/exp | 15324 | Adjusted field code and syntax., identical search term. |
| 2 | exp borderline state/dm, pc, rh, th [Disease Management, Prevention, Rehabilitation, Therapy] | 1945 | 2 | 'borderline state'/exp/dm_dm,dm_pc,dm_rh,dm_th | 2648 | Adjusted field code and syntax, identical search term. |
| 3 | (borderline and (personality or disorder)).ab. or (borderline and (personality or disorder)).ti. | 10336 | 3 | borderline:ab AND (personality:ab OR disorder:ab) OR (borderline:ti AND (personality:ti OR disorder:ti)) | 14424 | Adjusted field codes and syntax, identical search terms. |
| 4 | 1 or 3 | 14239 | 4 | #1 OR #3 | 19393 | Fully identical |
| 5 | exp psychiatric treatment/ | 300875 | 5 | ‘psychiatric treatment’/exp | 397307 | Adjusted field code and syntax, identical search term. |
| 6 | (‘transference focused psychotherapy’ or ‘schema-focused therapy’ or ‘dialectical behavior therapy’ or ‘mentalisation based treatment’ or ‘mentalization based treatment’).ab. or (‘transference focused psychotherapy’ or ‘schema-focused therapy’ or ‘dialectical behavior therapy’ or ‘mentalisation based treatment’ or ‘mentalization based treatment’).ti. | 737 | 6 | `transference focused psychotherapy’:ab OR ‘schema-focused therapy’:ab OR ‘dialectical behavior therapy’:ab OR ‘mentalisation based treatment’:ab OR ‘mentalization based treatment’:ab OR ‘transference focused psychotherapy’:ti OR ‘schema-focused therapy’:ti OR ‘dialectical behavior therapy’:ti OR ‘mentalisation based treatment’:ti OR ‘mentalization based treatment’:ti | 1266 | Adjusted field codes and syntax, identical search terms. |
| 7 | 5 or 6 | 300930 | 7 | #5 OR #6 | 397428 | Adjusted field code (#), identical syntax and search terms. |
| 8 | 4 and 7 | 3837 | 8 | #4 AND #7 | 5119 | Adjusted field code (#), identical syntax and search terms. |
| 9 | 2 or 8 | 4108 | 9 | #2 OR #8 | 5513 | Adjusted field code (#), identical syntax and search terms. |
| 10 | limit 9 to (meta analysis or "systematic review") | **113** | 10 | #9 AND ('meta analysis'/de OR 'systematic review'/de) | 223 | Adjusted field codes, adjusted syntax and identical search terms. |
|  |  |  |  | #10 AND [01-01-1900]/sd NOT [30-06-2017]/sd | **108** | Limited to date range of initial search |
| 11 | limit 9 to randomized controlled trial | **190** | 11 | #9 AND 'randomized controlled trial'/de | 338 | Adjusted field codes, adjusted syntax and identical search term. |
|  |  |  |  | #11 AND [01-01-1900]/sd NOT [30-06-2017]/sd | **182** | Limited to date range of initial search |

###

### Appendix B. Deduplication

Deduplication was done in two phases: (1) the automatic phase and (2) the manual phase.

#### Phase 1

In phase one, duplicates were automatically removed based on DOI, title and abstract using ASReview Datatools (De Bruin, 2020/2022). In this first phase, 480 duplicates were automatically detected and deleted. Afterward, our database consisted of k=1062 records.

#### Phase 2

In the second phase of deduplication, we manually took a look at the relevant studies to look for duplicates and equivalent papers which based their results on a same dataset. We deleted following records for the following reasons:

- (Bateman & Fonagy, 1999). Since 2017, the title of this article mentions: ‘Article did not do justice to available research data’. Therefore, we could view this article as a withdrawn article. Moreover, this initially relevant article changed a little regarding the title due to this issue. In our view, it was not ethically responsible to keep this article in the reconstructed database, because new information since the last search implies that one could not trust the outcomes of this study.
- (Giesen-Bloo et al., 2006a). This initially relevant study had two semi-duplicates: one correction record (Giesen-Bloo et al., 2006b) and one reply record (Giesen-Bloo & Arntz, 2007). To illustrate the decision process regarding these kinds of semi-duplicates, we mention four options we faced for these semi-duplicates and the final decision we took.
  1. Option one would be to label all these three articles as relevant. For the simulation-study model, this would, however, be problematic, because one paper will have three times more weight than relevant studies without any (semi-)duplicates.
  2. Option two would be to label the initially included study as relevant and the semi-duplicates as irrelevant. By using this option, the problem would be that almost the same titles and abstracts obtain different labels (i.e. relevant and irrelevant), which is extremely confusing to the simulation study model. How can it predict which studies are relevant when the same studies are labeled as both relevant and irrelevant?
  3. Option three is labeling the initially included study as relevant but leaving the semi-duplicates empty. In the NLF model presented in the next section, the empty labels could be screened. The problem with this option is that when leaving these records empty, these records will probably show up in the NLF model as probable relevant records and these will still be labeled relevant or irrelevant, which gives the same problems mentioned in options 1 and 2.
  4. The problems regarding the previous three options left us with no other choice than to choose option 4: deleting the semi-duplicates. It may lead to a somehow different database because we do not know if the initial dataset contained these semi-duplicates. However, this is the most feasible solution for a correct application of the NLF model and the simulation study described in the next sections.
- (McMain et al., 2009). This initially relevant study had one semi-duplicate in the form of a correction (McMain et al., 2010). According to the decision process described above, this semi-duplicate was deleted as well.
- (Turner, 2000a). This initially relevant study had one almost exact duplicate: a few words in the abstract differed and the author’s initials were not abbreviated in the duplicate (Turner, 2000b). Because it is an exact duplicate of the initial study, the second record by Turner (2000b) was deleted.
- (Linehan et al., 2015a). This initially relevant study had one semi-duplicate in the form of a correction (Linehan et al., 2015b). Again this semi-duplicate was removed.
- (Clarkin et al., 2007a). This initially relevant study had one almost exact duplicate: in the abstract, one record was with subheadings, and one record was without subheadings. Furthermore, the almost exact duplicate’s title had a lowercase letter after the colon (Clarkin et al., 2007b) instead of an uppercase letter in the record we assigned as initially relevant. Again the almost exact duplicate was deleted from the database.
- (Nadort et al., 2009b). This initially relevant study had one semi-duplicate in the form of a study design (Nadort et al., 2009a). Again this semi-duplicate was deleted.

Then there is the case of non-duplicates that only seem duplicates. The following records were not deleted, although they might seem similar to an initially relevant record.

- (Van den Bosch, 2005). This record seemed to be a Dutch-written version that has strong similarities with the initially included article (Van den Bosch et al., 2005). However, it was not deleted because it can be viewed as a different article, published in another journal.
- (McMain et al., 2012). This article seemed to be a follow-up study of the initially included article, published three years earlier (McMain et al., 2009). It was not deleted because of the same reason: it is a different article published at a different time with a different text.
- (Jorgensen et al., 2014). This article is a follow-up study of an initially included article, published one year earlier (Jorgensen et al., 2013). This follow-up study is not deleted because it is not a duplicate: there is different text and data in the follow-up study than in the initially included study.

### References used in the Appendix

Bateman, A. W., & Fonagy, P. (1999). Psychotherapy for severe personality disorder. Article did not do justice to available research data. *BMJ (Clinical Research Ed.)*, *319*(7211), 709–710; author reply 710-711.

Clarkin, J. F., Levy, K. N., Lenzenweger, M. F., & Kernberg, O. F. (2007a). Evaluating three treatments for borderline personality disorder: A multiwave study. *The American Journal of Psychiatry*, *164*(6), 922–928. https://doi.org/10.1176/appi.ajp.164.6.922

Clarkin, J. F., Levy, K. N., Lenzenweger, M. F., & Kernberg, O. F. (2007b). Evaluating three treatments for borderline personality disorder: A multiwave study. *The American Journal of Psychiatry*, *164*(6), 922–928. https://doi.org/10.1176/ajp.2007.164.6.922

De Bruin, J. (2022). *ASReview Datatools* [Python]. ASReview. https://github.com/asreview/asreview-datatools (Original work published 2020)

Giesen-Bloo, J., & Arntz, A. (2007). ‘Outpatient psychotherapy for borderline personality disorder: A randomized trial of schema-focused therapy vs transference-focused psychotherapy’: Reply. *Archives of General Psychiatry*, *64*(5), 610–611. https://doi.org/10.1001/archpsyc.64.5.610

Giesen-Bloo, J., Van Dyck, R., Spinhoven, P., Van Tilburg, W., Dirksen, C., Van Asselt, T., Kremers, I., Nadort, M., & Arntz, A. (2006a). Outpatient Psychotherapy for Borderline Personality Disorder: Randomized Trial of Schema-Focused Therapy vs Transference-Focused Psychotherapy. *Archives of General Psychiatry*, *63*(6), 649–658. https://doi.org/10.1001/archpsyc.63.6.649

Giesen-Bloo, J., Van Dyck, R., Spinhoven, P., Van Tilburg, W., Dirksen, C., Van Asselt, T., Kremers, I., Nadort, M., & Arntz, A. (2006b). ‘Outpatient Psychotherapy for Borderline Personality Disorder: Randomized Trial of Schema-Focused Therapy vs Transference-Focused Psychotherapy’: Correction. *Archives of General Psychiatry*, *63*(9), 1008. https://doi.org/10.1001/archpsyc.63.9.1008

Internet archive (archive.org). (2017, July 5). *Systematic Reviews Subset Strategy*. Systematic Reviews Subset Strategy. https://web.archive.org/web/20170705092445/https://www.nlm.nih.gov/bsd/pubmed_subsets/sysreviews_strategy.html

Jorgensen, C. R., Boye, R., Andersen, D., Dossing Blaabjerg, A. H., Freund, C., Jordet, H., & Kjolbye, M. (2014). Eighteen months post-treatment naturalistic follow-up study of mentalization-based therapy and supportive group treatment of borderline personality disorder: Clinical outcomes and functioning. *Nordic Psychology*, *66*(4), 254–273. https://doi.org/10.1080/19012276.2014.963649

Jorgensen, C. R., Freund, C., Boye, R., Jordet, H., Andersen, D., & Kjolbye, M. (2013). Outcome of mentalization-based and supportive psychotherapy in patients with borderline personality disorder: A randomized trial. *Acta Psychiatrica Scandinavica*, *127*(4), 305–317. https://doi.org/10.1111/j.1600-0447.2012.01923.x

Linehan, M. M., Korslund, K. E., Harned, M. S., Gallop, R. J., Lungu, A., Neacsiu, A. D., McDavid, J., Comtois, K. A., & Murray-Gregory, A. M. (2015a). Dialectical behavior therapy for high suicide risk in individuals with borderline personality disorder: A randomized clinical trial and component analysis. *JAMA Psychiatry*, *72*(5), 475–482. https://doi.org/10.1001/jamapsychiatry.2014.3039

Linehan, M. M., Korslund, K. E., Harned, M. S., Gallop, R. J., Lungu, A., Neacsiu, A. D., McDavid, J., Comtois, K. A., & Murray-Gregory, A. M. (2015b). ‘Errors in continuing medical education questions and answers’: Correction. *JAMA Psychiatry*, *72*(9), 951. https://doi.org/10.1001/jamapsychiatry.2015.1480

McMain, S. F., Guimond, T., Streiner, D. L., Cardish, R. J., & Links, P. S. (2012). Dialectical behavior therapy compared with general psychiatric management for borderline personality disorder: Clinical outcomes and functioning over a 2-year follow-up. *The American Journal of Psychiatry*, *169*(6), 650–661. https://doi.org/10.1176/appi.ajp.2012.11091416

McMain, S. F., Links, P. S., Gnam, W. H., Guimond, T., Cardish, R. J., Korman, L., & Streiner, D. L. (2009). A randomized trial of dialectical behavior therapy versus general psychiatric management for borderline personality disorder. *The American Journal of Psychiatry*, *166*(12), 1365–1374. https://doi.org/10.1176/appi.ajp.2009.09010039

McMain, S. F., Links, P. S., Gnam, W. H., Guimond, T., Cardish, R. J., Korman, L., & Streiner, D. L. (2010). ‘A randomized trial of dialectical behavior therapy versus general psychiatric management for borderline personality disorder’: Correction. *The American Journal of Psychiatry*, *167*(10), 1283.

Nadort, M., Arntz, A., Smit, J. H., Giesen-Bloo, J., Eikelenboom, M., Spinhoven, P., van Asselt, T., Wensing, M., & van Dyck, R. (2009a). Implementation of outpatient schema therapy for borderline personality disorder: Study design. *BMC Psychiatry*, *9*(American Psychiatric Association. (2005). Diagnostic and statistical manual of mental disorders, text revision Fourth edition. Washington, DC: American Psychiatric Association; 2005.). https://doi.org/10.1186/1471-244X-9-64

Nadort, M., Arntz, A., Smit, J. H., Giesen-Bloo, J., Eikelenboom, M., Spinhoven, P., van Asselt, T., Wensing, M., & van Dyck, R. (2009b). Implementation of outpatient schema therapy for borderline personality disorder with versus without crisis support by the therapist outside office hours: A randomized trial. *Behaviour Research and Therapy*, *47*(11), 961–973. https://doi.org/10.1016/j.brat.2009.07.013

National Library of Medicine. (2019, February 20). *Search Strategy Used to Create the PubMed Systematic Reviews Filter* [Training Material and Manuals]. Search Strategy Used to Create the PubMed Systematic Reviews Filter; U.S. National Library of Medicine. https://www.nlm.nih.gov/bsd/pubmed_subsets/sysreviews_strategy.html

Turner, R. M. (2000a). Naturalistic evaluation of dialectical behavior therapy-oriented treatment for borderline personality disorder. *Cognitive and Behavioral Practice*, *7*(4), 413–419. Embase. https://doi.org/10.1016/S1077-7229(00)80052-8

Turner, R. M. (2000b). Naturalistic evaluation of dialectical behavior therapy-oriented treatment for borderline personality disorder. *Cognitive and Behavioral Practice*, *7*(4), 413–419. https://doi.org/10.1016/S1077-7229%2800%2980052-8

Van den Bosch, L. M. C. (2005). Efficacy of dialectical behaviour therapy in the treatment of female borderline patients with and without substance abuse problems: Result of a Dutch study. *Dialectische gedragstherapie bij Nederlandse vrouwen met een borderline persoonlijkheidsstoornis, met en zonder verslavingsproblemen.*, *47*(3), 127–137.

Van den Bosch, L. M. C., Koeter, M. W. J., Stijnen, T., Verheul, R., & Van den Brink, W. (2005). Sustained efficacy of dialectical behaviour therapy for borderline personality disorder. *Behaviour Research and Therapy*, *43*(9), 1231–1241. https://doi.org/10.1016/j.brat.2004.09.008

1. Due to an error, #results were not reported for each query at 1-11-2022. Because of that, the search was performed again 19-01-2023. The exported query S15 yielded the same amount of results as 1-11-2022 (41), S13 with date correction yielded one record less: 524 results at 19-01-2023 instead of 525 at 1-11-2022.   [↑](#footnote-ref-2)
